# Assessment of body composition by dual-energy X-Ray absorptiometry in renal transplant patients, hemodialysis patients, and a control group of healthy subjects

**DOI:** 10.1016/j.clinsp.2024.100505

**Published:** 2024-09-27

**Authors:** Martha Jocelyne Piñon-Ruiz, Maria-Raquel Huerta-Franco, Francisco-Miguel Vargas-Luna, Evelia Apolinar-Jimenez, Joel Máximo Soel Encalada

**Affiliations:** a Division of Medical Sciences, University of Guanajuato-Campus León, Gto, Mexico; b Department of Sciences Applied to Work, Division of Health Sciences, University of Guanajuato – Campus León, Gto, Mexico; c Department of Physical Engineering, Division of Sciences and Engineering, University of Guanajuato – Campus Leon, Gto, Mexico; d Metabolism and Nutrition Unit, Regional Hospital of High Specialty of Bajio, Instituto Mexicano del Seguro Social para el Bienestar, Mexico; e Health Department, Regional Hospital of High Specialty of Bajio, Transplant Unit, Gto, Mexico

**Keywords:** Body composition, DEXA scan, Chronic kidney disease, Kidney transplantation

## Abstract

**Background:**

The clinical findings of patients with Chronic Kidney Disease (CKD), which is characterized by malnutrition, sedentary lifestyle, uremia, and catabolism associated with dialysis produce changes in Body Composition (BC), causing increased Fat Mass (FM), decreased in both Lean Mass (LM) and Body Mineral Density (BMD), even despite uremic reversal after a Kidney Transplant (KT); immunosuppressive medications alter BC, increasing the risk of loss of the kidney transplant and cardiovascular diseases.

**Objective:**

To demonstrate whether there are differences in BC between a group of patients with KT and a group of patients on Hemodialysis (HD), when comparing them with a control group without the disease.

**Materials and methods:**

In the present observational study, with a comparative design; 125 patients were evaluated (46 with KT, 47 on HD, and 32 from the healthy control group). The BC was evaluated with the full-body Dual-Energy X-Ray Absorptiometry (DEXA) method.

**Results:**

The mean age and standard deviation (X±SD) of the study subjects were: 28.89 ± 5.76, 27.39 ± 5.04, and 29.63 ± 6.34 years for the HD, KT, and control subjects, respectively. The HD patients presented a total FM of 14.98 ± 6.96 kg in comparison with 20.1 ± 6.5 kg for the control group (p = 0.007), and 19.06 ± 7.94 kg for the group with KT (p = 0.02). The total LM was lower in the KT patients in comparison with the control group (p = 0.023). The content and total BMD were lower in both groups of patients with KT and HD.

**Conclusions:**

Although a comprehensive improvement in BC was expected after kidney transplantation, the results are not close to “normal’ values, when compared with those of healthy subjects of the same age.

## Highlights

• The body fat of patients with kidney transplants was higher when compared to patients on hemodialysis and the control group.
• Bone mineral content and bone mineral density were similar in hemodialysis patients and kidney transplant patients.
• Body composition assessment in patients with chronic kidney disease could be an aid in their clinical control.

## Introduction

CKD has high mortality and disability; the prevalence rates of CKD are increasing day by day worldwide.^1^ In Mexico, a prevalence of 12.2% has been reported; and there is a rate of 51 deaths per 100 thousand inhabitants.^2^ Although Mexican patients with CKD would be expected to present an optimal body composition (with an increase in lean mass and bone mass) after kidney transplant; it has been shown that their body composition is not close to “normal” values, when compared with the body composition of healthy subjects of the same age.^3^, ^4^^,^^5^ Despite the reversal of uremia after KT, immunosuppressive medications produce changes in patients’ BC; for example, weight gain, and a decrease in lean mass.^6^, ^7^, ^8^, ^9^, ^10^ The above is associated with cardiovascular diseases, and with lower patient and kidney graft survival.^11^^,^^12^ Therefore, it is important to investigate the BC of patients with CKD, those with KT, and those who are on HD, to see the effects of replacement treatments on BC, and in this way conduct treatment and rehabilitation programs that produces excellent physical function in this group of patients, and thereby reduce graft losses and associated comorbidities.^3^^,^^10^^,^^13^^,^^14^ Therefore, the objective of the present study was to determine BC using the DEXA technique in a group of patients with CKD (one group with KT, and another on HD), and compare the results with those of a group of healthy participants.

## Materials and methods

The present observational research with a cross-sectional, and analytical design was made following the STROBE statement. This investigation was approved by the Research and Research Ethics Committees of the Regional Hospital of High Specialty of Bajio, Guanajuato, Mexico with the following registration numbers: CI/HRAEB/018/2022 and CEI-010-2022

### Subjects

Forty-seven adult patients with CKD undergoing periodic hemodialysis; 46 patients with kidney transplants, and 32 healthy participants (made up of the control group) were evaluated.

The group of HD patients met the following inclusion criteria: 1) Having a minimum of three months and a maximum of 60 months of dialysis treatment, with a frequency of two to three treatment sessions per week; 2) present a stable state of health condition with serum hemoglobin levels > 6.3 mM/L, subjected to a normal protein diet, and 3); to be treated with pharmacological therapy according to their protocol, and with appropriate management for any comorbidity they presented.

The group of patients with KT met the following inclusion criteria: 1) Having a minimum of three months and a maximum of 60 months since the transplant (this time was determined because the consequences of immunosuppressants and physical inactivity mainly on the content of fat mass and lean mass after the transplant can be seen a month later, reaching its peak at 12 months);^15^ 2) be treated with standard maintenance immunosuppression, according to the protocols of the hospital where the transplant was performed (tacrolimus/cyclosporine combined with mycophenolic acid, and methylprednisolone), 3) present serum levels of creatinine, and urea no higher than 2.90 mg/dL, and 160 mg/dL respectively.

For this research, “healthy” control group subjects are defined as participants who met the following inclusion criteria: 1) Being adults over 18 years of age; 2) that they did not present CKD or any previously diagnosed chronic or acute disease (except for overweight or obesity since the subjects in the control group were matched according to the BMI of the study groups) that affected the study variables; 3) be relatives of the KT patients, and be relatives of the HD patients in this study (to avoid socioeconomic level and lifestyle biases between the three groups); 4) being sedentary people (that is, not doing regular exercise as indicated by the American College of Sports Medicine);^16^ a total of 32 subjects met the inclusion criteria previously indicated.

Patients with KT who had a history of acute KT rejection proven by a biopsy performed in the last three months prior to inclusion in the study were excluded; if they had any musculoskeletal problem that prevented independent ambulation; if they had cognitive deficits; amputation of extremities; history of hospitalizations in the last month; previous clinical history of heart diseases, or hemodynamically unstable: atrial fibrillation, heart failure, coronary artery disease, pulmonary embolism, aortic aneurysm, dilated cardiomyopathy, hypertrophic cardiomyopathy, acute myocardial infarction, and hypertension); and if they had a diagnosis of systemic lupus erythematosus or diabetes mellitus.

### Anthropometric assessment

The principal investigator made all anthropometric evaluations that included body weight (kg), height (cm), and body mass index (BMI, kg/m^2^) using the standard procedure;^17^ and measured the weight and height with a digital scale which had an integrated stadiometer (Seca®, model 763). For HD patients, body weight was assessed 3 to 5 hours after the dialysis session.

### Procedure in assessing Body Composition (BC)

BC was determined by whole-body Dual-Energy X-Ray Absorptiometry (DEXA) using a Hologic Discovery Wi® device. Fat Mass (FM), Lean Mass (LM), percentage of body Fat (%FM), and Lean body Mass (%LM), Bone Mineral Density (BMD), and total bone mineral content were measured of the lower limbs, upper limbs, and trunk; in addition, the total T-score was calculated. In HD patients, to control overhydration, all patients were evaluated within 3 to 5 hours after their hemodialysis session; blood pressure also was measured (Welch Allyn ds-44-11baumanometer and Littmann 5863 stethoscope) and it was determined that these were within normal parameters (< 129 mm.Hg., for the systolic blood pressure and < 85 mm.Hg for the diastolic blood pressure); likewise, in the clinical examination, the absence of edema was determined.

### Laboratory data

The serum levels of hemoglobin, creatinine, albumin, urea, blood urea nitrogen, and the electrolytes sodium, potassium, calcium, magnesium, and phosphorus were determined. The principal researcher assessed the renal function of all patients considering the Glomerular Filtration Rate (GFR) and using the formula according to the CKD-EPI.^18^

### Statistical analysis

The authors performed the statistical analysis of the data using SPSS 25.0 software (SPSS Inc., Chicago Illinois). The qualitative variables (sociodemographic and clinical variables) were presented in absolute and relative frequency tables. These variables were compared between the groups with χ^2^ tests. The authors also used the Shapiro-Wilk test to determine the type of distribution of all quantitative variables. The variables with normal distribution are presented with values of average ± Standard Deviation (X±SD). Variables without normal distribution are presented with median values and interquartile ranges. The differences between continuous and normally distributed variables between the three groups were compared using one-way analysis of variance (ANOVA) and performed post-hoc range tests (Bonferroni test). Variables without normal distribution between groups were compared with non-parametric Kruskal-Wallis H statistical tests and Mann-Whitney *U* tests to compare pairwise medians. In all statistical analyses the authors consider a significant value when p < 0.05.

## Results

### Differences in demographic and clinical characteristics between HD patients, patients with KT, and healthy control subjects

One hundred and twenty-five volunteers participated in this research (47 in the HD group, 46 in the KT group, and 32 in the control group). The KT, HD, and control groups were composed of a higher percentage of men 25 (53.2%) in the HD group, 25 (54.3%) in the KT group, and 19 (59.4%) in the control group. The results of the χ^2^ tests did not show significant differences when comparing these frequencies, thus having greater control over the anthropometric differences between sexes.

When comparing the average age between the three groups of participants, no significant differences were observed, with the results being 28.89±5.76 years for the HD group; 27.39 ± 5.04 years for the KT group; and 29.63 ± 6.34 years for the control group (Table 1). The average time since the patients' diagnosis of CKD (p < 0.001), and the start of dialysis sessions in the HD group was significantly shorter when compared to the time since surgery in the KT group (p = 0.016) (Table 2). The history of performing physical activity on a regular basis according to the recommendations of the American College of Sports Medicine,^16^ was significantly higher in the control group, compared to the HD and KT groups (p < 0.001). In this research, the authors also observed that the frequencies of participants who had a job were higher in the control group compared to the HD and KT groups (p < 0.001). It is important to note that there were no significant differences between the latter two (Table 1).

**Table 1:** Differences in sociodemographic characteristics between patients on HD, patients with KT, and the control group of healthy subjects.

	Hemodialysis (HD) (n = 47)	Kidney transplant (KT) (n = 46)	Healthy control (n = 32)	p-value
**Age (years)**	28.89 ± 5.76	27.39 ± 5.04	29.63 ± 6.34	0.202
**Sex**				0.850
Men	25 (53.2%)	25 (54.3%)	19 (59.4%)	
Women	22 (46.8%)	21 (45.7%)	13 (40.6%)	
**Job occupation**	19 (40.4%)	28 (60.90%)	29 (90.6%)^a^	**<0.001**
**Physical activity**	3 (6.4 %)	3 (6.5%)	14 (43.8%)^b^^,^^c^	**<0.001**
**Scholarship**				0.242
Elementary school	7 (14.9%)	8 (17.4%)	1 (3.1%)	
Middle school	24 (51.1%)	22 (47.8%)	13 (40.6%)	
High school	12 (25.5%)	9 (19.6%)	13 (40.6%)	
Bachelor	4 (8.5%)	7 (15.2%)	5 (15.6%)	
Adherence to a dietary regimen	5 (10.6%)	8 (17.4%)	1 (3.1%)	0.143
**Hypertension**	39 (83%)	24 (52.25%)	‒	**0.002**

AVF, Arteriovenous Fistula, qualitative variable reported as frequency and percentage, quantitative variable with normal distribution, presented as mean and standard deviation; quantitative variable without normal distribution, such as median and interquartile ranges.

Bonferroni adjustment.

a p = 0.001 vs. HD.

b p = 0.001 vs. KT

c p = 0.001 vs. HD.

**Table 2:** Differences in clinical characteristics of chronic kidney disease patients.

	Hemodialysis (HD) (n = 47)	Kidney transplant (KT) (n = 46)	p-value
**Hypertension**	39 (83%)	24 (52.25%)	**0.002**
**Etiology of CKD**			**0.122**
Idiopathic	39 (82.9%)	40 (87%)	
Others	8 (17.1%)	6 (13%)	
**Time since CKD diagnosis (months)**	30 (16‒60)	72 (69‒107.25)	**<0.001**
**Time with KT or HD (months)**	19 (14‒35)	41 (15.75‒49.75)	**0.016**
**Donor type**			
Deceased	‒	18 (39.13%)	
Alive	‒	28 (60.87%)	
**Vascular access (HD)**			
AVF	15 (31.9%)		
Niagara catheter	5 (10.6%)		
Mahurkar catheter	7 (14.9%)		
Permacath	20 (42.6%)	‒	
**Medication**			
Cyclosporin	‒	32 (70%)	
Tacrolimus	‒	14 (30%)	
Mycophenolic acid	‒	46 (100%)	
Prednisone	‒	46 (100%)	
Beta-blockers	39 (83%)	24 (52.25%)	**0.002**

AVF, Arteriovenous Fistula, quantitative variable with normal distribution, presented as mean and standard deviation; quantitative variable without normal distribution; as median and interquartile ranges.

### Laboratory data results between HD patients and KT patients

Patients with KT had significantly lower levels of serum creatinine < 0.0001, urea < 0.0001, blood urea nitrogen < 0.0001, magnesium < 0.0001, phosphorus < 0.0001, and potassium < 0.0001) than the HD group. Regarding GFR, it was higher in the KT group < 0.0001, as well as hemoglobin concentration < 0.0001, and calcium < 0.0001 in comparison with patients on HD; however, albumin levels were similar in both groups (0.88) (Table 3).

**Table 3:** Differences in the laboratory results of patients with chronic kidney disease.

	Hemodialysis (HD) (n = 47)	Kidney transplant (KT) (n = 46)	p-value
**Urea nitrogen (BUN) (mg/dL)**	63 (51.0‒77.0)	22 (18.0‒31.0)	**<0.0001**
**Urea (mg/dL)**	134.80 (109.10‒159.45)	47.10 (38.50‒62.10)	**<0.0001**
**Creatinine (mg/dL)**	12.40 (9.30‒16.50)	1.6 (1.20‒1.90)	**<0.0001**
**Hemoglobin (g/dL)**	10.53 ± 1.85	13.35 ± 3.04	**<0.0001**
**Albumin (g/dL)**	4.3 (3.95‒4.50)	4.3 (4.20‒4.60)	**<**0.879
**Magnesium (mg/dL)**	2.4 (2.10‒2.85)	2 (1.70‒2.20)	**<0.0001**
**Phosphorus (mg/dL)**	5.9 (5.05‒7.50)	3.9 (3.40‒4.20)	**<0.0001**
**Potassium (mmoL/L)**	5.6 (4.70‒6.10)	4.5 (4.30‒4.80)	**<0.0001**
**Calcium (mg/dL)**	8.7 (7.80‒9.30)	9.5 (9.2‒10.10)	**<0.0001**
**GFR (mL/min/1.73 m^2^)**	4.07 (3.27‒5.40)	61.88 (49.98‒72.36)	**<0.0001**

GFR, Glomerular Filtration Rate, qualitative variable reported as frequency and percentage, quantitative variable with normal distribution, presented as mean and standard deviation (X±SD); quantitative variable without normal distribution, such as median and interquartile ranges, p values in bold indicate statistical significance (p≤0.05).

### Results of anthropometric characteristics and body composition among HD patients, KT patients compared to the control group

Body weight was significantly higher in the control group when compared with the two CKD groups (of HD and KT, p = 0.02). BMI values were significantly higher in the control group in comparison to the group of patients on HD, but were like those in the KT group. However, the percentages of overweight and obesity were similar when comparisons were made between the three groups (Table 4).

**Table 4:** Differences in anthropometric characteristics and body composition between patients on Hemodialysis (HD), patients with Kidney Transplant (KT) and the control group.

	Hemodialysis (n = 47)	Kidney transplant (n = 46)	Control group (n = 32)	p-value
**Weight (kg)**	63.23 ± 13.94	63.52 ± 13.54	71.4 ± 14.32^a,^^b^	**0.020**
**Height (cm)**	161.67± 8.40	159.54 ± 9.04	163.5 ± 10.8	0.172
**BMI (kg/m^2^)**	22.9 (21.25‒26.5)	23.1 (21.25‒23.1)	26.49 (23.7‒29.05)^c^	**0.016**
**Obesity/overweight**	16 (34%)	17 (36.9%)	16 (50%)	0.335
**Total fat mass (kg)**	14.98±6.96^d^^,^^e^	19.06± 7.94	20.1±6.5	**0.004**
**Total lean mass (kg)**	43.92 ± 10.32	41.0 ± 8.02^f^	47.11 ± 11.73	**0.031**
**Total bone mineral density (BMD) (g/cm^2^)**	0.99 ± 0.10	1.01 ± 0.10	1.12 ± 0.10^g^^,^^h^	**<0.001**
**Total bone mineral content (BMC) (kg)**	1.83 ± 0.38	1.86 ± 0.37	2.23 ± 0.49^g^^,^^h^	**<0.001**
**Lean mass + BMC (kg)**	45.72 ± 10.5	50.25 ± 49.47	49.39 ± 12.16	0.765
**% Total fat**	24.88 ±8.49 i	30.06 ± 8.60	29.24 ±8.53	**<0.011**
**T score**	‒1.60 ± 1.27	‒1.32 ± 1.2	‒0.96 ± 0.95^j^^,^^k^	**<0.001**

Bonferroni posthoc analysis, pairwise comparison ^a^ p = 0.029 vs. HD

b p = 0.038 vs. KT

c p = 0.16 vs. HD

d p = 0.02 vs. KT

e p = 0.007 vs. healthy-control

f p = 0.023 vs. healthy control

g p = 0.001 vs. HD

h p = 0.001 vs. KT

i p = 0.011 vs. KT

j p = 0.001

k p = 0.001.

**Fig. 1:**
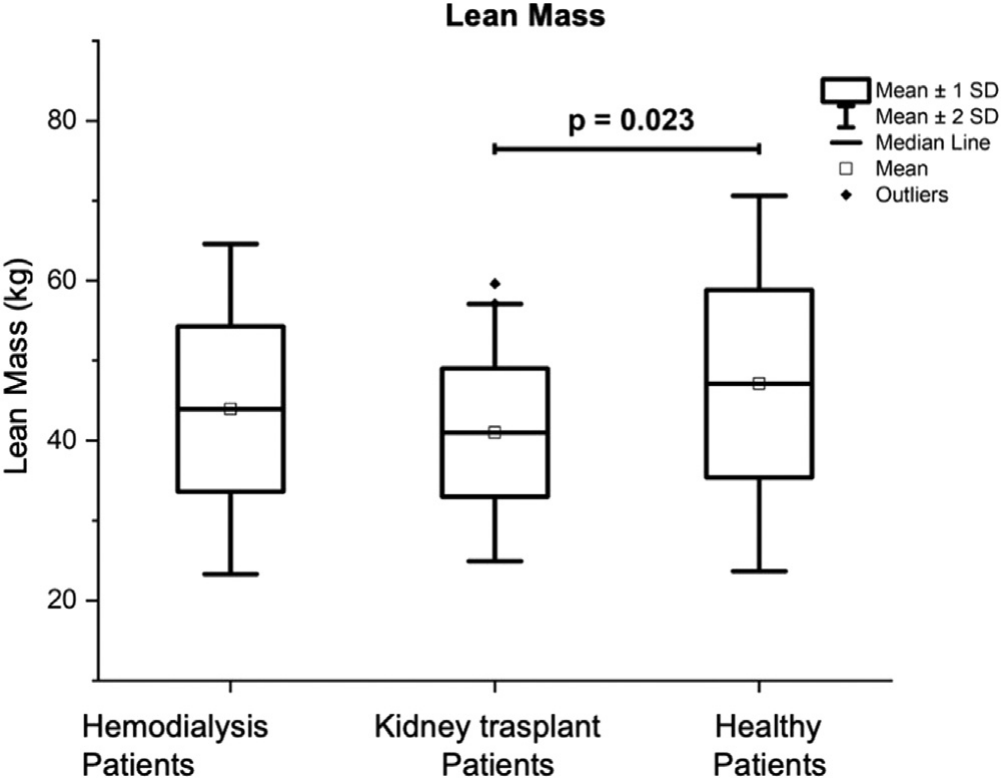
Comparison of lean mass (in kg) of patients with kidney transplant, patients on hemodialysis and the control group.

**Fig. 2:**
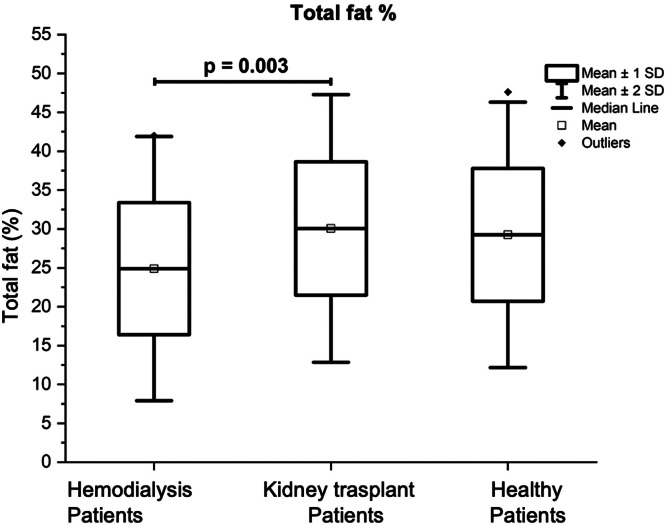
Comparison of total body fat percentage (considering body weight as 100%) of kidney transplant patients, hemodialysis patients, and the control group.

Table 4 also presents the results of the Total Fat Mass (TFM) of the three groups of volunteers which was significantly lower in the group of patients on HD (14.98 ± 6.96 kg) in comparison with the other two groups, being the results of: 19.06 ± 7.94 kg for the group with KT (p = 0.02), and 20.1 ± 6.5 kg for the control group (p = 0.007). Patients with KT had a significantly lower Total Lean Mass (TLM) (41.0 ± 8.02 kg) in comparison with the control group (47.11 ± 11.73 kg; p = 0.023) (see Fig. 1). The percentage of body total fat was significantly higher in the patients with KT (30.06% ± 8.60) in comparison with the patients on HD (24.88% ± 8.49; p = 0.01); see Fig. 2. Table 4 also shows that Bone Mineral Density (BMD), and Bone Mineral Content (BMC) were significantly higher in the control group, in comparison with patients on HD, and patients with KT (p < 0.05).

Table 4 also presents the results of the Total Fat Mass (TFM) for the upper extremities of the three groups of volunteers; this was significantly lower in the group of HD patients (14.98 ± 6.96 kg) compared to the other two groups (19.06 ± 7.94 kg). The BMD results were significantly lower in the groups with CKD (patients on HD, and patients with KT), with the results being as follows: 0.71 ± 0.08 g/cm^2^ for the group with HD (p < 0.001); and 0.72 ± 0.08 g/cm^2^ for the group of patients with KT, and 0.78 ± 0.08 g/cm^2^, for the control group, respectively. Furthermore, the median and interquartile range values of BMD in the upper limbs of patients with KT were significantly lower, when compared to those of the other groups, the results being as follows: 0.13 (0.11‒0.16) kg compared to the control group, the results being: 0.16 (0.13‒0.2) kg; p = 0.04.

**Fig. 3:**
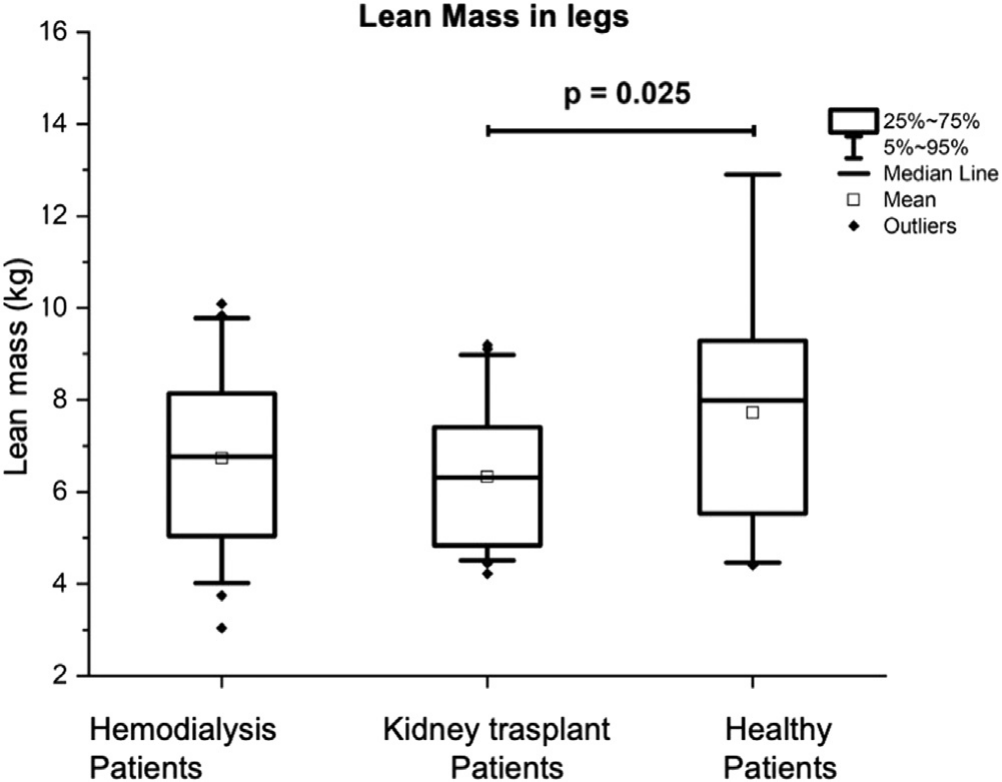
Comparison of lean mass (in kg) of patients with kidney transplant, patients on hemodialysis and the control group.

In the lower extremities, the LM of the group with KT was significantly lower 6.31 (4.83‒7.5 kg) compared to that of the control group, which was 7.99 (5.48‒9.36 kg), (p = 0.025) (see Fig. 3). The BMD was significantly higher in the control group with 1.19 (1.01‒1.32) g/cm^2^, in comparison with the patients on HD being of 1.00 (0.92‒1.12) g/cm^2^ (p = 0.006), and being of 1.19 (1.01‒1.32) g/cm^2^ for the KT patients (p = 0.018). The BMC was also significantly higher in the control group being 0.32 (0.27‒0.39) kg in comparison with the patients with KT, being the results of 0.45 (0.31‒0.51 kg), (p = 0.006), and 0.33 (0.28‒0.39) kg for the HD patients (p < 0.001).

When comparing the FM in the trunk found that it was significantly lower in the HD patients when compared with that of the KT patients and the control group, with the “p” values being (p = 0.018 and p < 0.001, respectively). The BMC and total trunk fat percentage results of the control group were higher compared to those of the chronic kidney disease patient groups (HD and KT patients), see Table 5.

**Table 5:** Differences in the trunk body composition, between patients on hemodialysis, patients with kidney transplant and the control group.

	Hemodialysis (n = 47)	Kidney transplant (n = 46)	Control group (n = 32)	p-value
**Trunk fat mass (kg)**	5.78 (3.63‒8.88)^a,^^b^	8.17 (5.25‒12.38)	9.73 (7.93‒12.09)	**<0.001**
**Trunk lean mass (kg)**	21.70 (17.35‒25.40)	19.10 (16.92‒23.15)	23.42 (16.55‒26.40)	0.075
**Trunk bone mineral content (BMC) (kg)**	0.44 ± 0.10	0.44 ± 0.10	0.58 ± 0.13^c^^,^^d^	**<0.001**
**Total % trunk fat.**	23.01 ± 9.11^f^^,^^g^	29.38 ± 9.47	30.36 ± 7.83	**<0.001**

Bonferroni post-hoc analysis, pairwise comparison: ^a^ p = 0.018 vs. KT

b p = 0.001 vs. healthy-control

c p = 0.001 vs. HD

d p = 0.001 vs. KT

f p = 0.002 vs. KT

g p = 0.001 vs. healthy-control.

To determine the influence of time on hemodialysis or kidney transplant on body composition variables, each of the groups was divided into 4 specific subgroups (< 12 months, 13‒24 months, 25‒48 months and > 48-months; and a comparison was made between these subgroups). The result showed that in the HD group, BMD was lower in the 12-month subgroup compared to the > 48-month subgroup on hemodialysis (1.05 ± 0.07 vs. 0.90 ± 0.10) (p = 0.03). Furthermore, the Pearson correlation test demonstrated a correlation coefficient of *r* = -0.51 (p < 0.001). The kidney transplant group was divided into 4 subgroups (similar to what was done in the HD group); the results showed no statistically significant differences between the subgroups in any of the body composition variables.

In the same way, the possible correlations of the body composition variables with the time since the diagnosis of CKD were analyzed in both groups, resulting in a correlation coefficient of the variable time since the diagnosis and the percentage of fat-free mass in the arms *r* = -0.31 (p = 0.033); and the arm fat percentage *r* = 0.31 (p = 0.035) only in the kidney transplant group.

## Discussion

The results of this research demonstrated that HD patients presented lower figures in the results of total body fat mass, trunk fat mass, and total trunk fat weight percentage when compared to those in the control group, and with those of patients with KT. TFM and lower extremity lean mass were significantly lower in KT patients compared with those in the control group.

When comparing the results of BMD content, these were significantly lower in HD patients and in KT patients. These results were similar to those found by other researchers who evaluated changes in WC in patients with KT, although in those studies the researchers used the bioelectrical impedance technique.^18^, ^19^, ^20^

In a longitudinal study, which was conducted over 24 months, Dienemann et al.^21^ evaluated the QoL of patients with KT and compared it with that of a group of healthy controls; however, the average age was different between the groups, being 46 years (range of 20‒60) and 39 years (range of 21‒60) for the group of patients with KT, and for the control group, respectively. The researchers demonstrated that there was a significant increase in the body weight of patients with KT, with the average values being 86.7 (48.4‒135.3) kg vs. 74.7 (41.5‒123.5) kg. These researchers also demonstrated decreased FM in the upper and lower extremities of KT patients; they observed that 45% of the patients were obese at 24 months of follow-up.^21^ In the present study, the time elapsed since the date of surgery for patients with KT was 41 months; the prevalence of overweight and obesity (BMI, kg/m^2^) in this group of patients was 36.9%; figures like those reported by Dienemann et al.^21^ However, the authors did not find significant differences when comparing FM between patients with KT, patients on HD, and the control group. These results can be explained by the high prevalence of overweight and obesity (50%) in the control group subjects evaluated in this research. In this research, the results of total fat mass were similar between the groups, coinciding with the results of other studies, which demonstrate the increase in body weight in patients with KT at the expense of adipose tissue.^19^

In various studies it has been shown that patients with KT present a decrease in lean mass (due to the loss of muscle mass in the lower limbs); the researchers show that there is a loss of muscle mass of approximately 10% in the lower limbs, compared to the healthy control group. An increase in trunk FM was also observed in KT patients treated with cyclosporine.^22^, ^23^, ^24^ These results coincide with those reported in this research, since the authors found a lower BF in the lower limbs of patients with KT when compared to patients in the control group, being 20.9% lower for the former. This study also showed that 70% of patients with KT were receiving cyclosporine, which suggests that the immunosuppressive therapy received by patients with KT may influence the BC and the distribution of body fat.

This study also showed that there was lower BMD in the upper limbs (especially the arms) of patients with CKD; results that were like those reported by Kang et al.^25^ The differences in BMD, observed in the study patients and by other researchers, can be explained by the following reasons: 1) By the decrease in lean mass, 2) by the prolonged consumption of calcineurin inhibitors in the patients with KT, and 3) due to the physical inactivity characteristic of Mexican patients with CKD; since in most health systems in Mexico, physical conditioning program protocols are not followed for patients with CKD.^26^, ^27^, ^28^, ^29^ In the scientific literature, results like those found in the present investigation have been reported in the BMD figures of patients with KT; and those observed in HD patients. Therefore, it is concluded that patients on HD and those with KT have lower BMD when compared to those in the control group.^20^, ^21^, ^22^, ^30^

The results of the most recent literature on the study of body composition in patients with CKD allow us to point out that there are some difficulties in making comparisons of these studies with those found in the present investigation due to the following reasons: i) Due to the different methodological designs in each of the studies; ii) due to the different sample sizes of patients evaluated; iii) due to the different evolution times of patients on HD and with KT; iv) even more so due to the different methodologies used to evaluate body composition; v) due to cultural differences in eating habits and customs, and in the practice of physical exercise of patients from developing countries with CKD. In addition to the above, the studies in which the body composition of patients with CKD has been evaluated come from Europe and Asia, which are countries in which the BMI of their patients with KT is notably lower when compared with the BMI of their patients.^31^, ^32^, ^33^ Even more, the Mexican population of patients on HD and with KT has a much lower average age (28.89±5.76, and 27.39±5.04, years for patients on HD and with KT, respectively), when compared with the average age reported for CKD patients from the aforementioned studies (average age 50 years); so low lean mass; and increased fat mass in patients studied in Europe and Asia could also be related to advanced age.^23^, ^24^, ^25^, ^26^, ^34^, ^35^

This investigation has some limitations such as the probable bias in the selection of CKD patients; since patients with severe physical disabilities and graft loss were not included; therefore, body composition might have been overestimated. In this investigation, CKD patients were also selected from a single hospital; therefore, a larger sample of patients from various hospitals is required; the problem that could result in a limitation when generalizing the results to Mexican patients with KCD. However, this is the first research with a comparative design carried out in Mexican patients on HD and with KT in which a highly valid and sensitive technique was used to measure body composition, so it is expected that this study will be a starting point for further investigations. Therefore, more studies are required in which patients from different hospitals are evaluated, and longitudinal designs are carried out to see the effect of physical conditioning programs on improving the quality of life and the survival rate of patients with KT and those on hemodialysis.

## Conclusions

The results of the present investigation demonstrate that although patients with KT should have a body composition like that of a healthy control; the body composition of these patients shows alterations similar to those observed in HD patients, and some are even worse, such as a decrease in lean mass. This research also showed that patients with CKD presented alterations in body composition when compared to patients in the control group. Therefore, it is advisable to carry out more studies with prospective designs to evaluate the evolution of body composition in patients after kidney transplantation. Finally, in the future, it is proposed to develop strategies applying physical conditioning programs for patients with CKD, in this way, it will be possible to improve the quality of life and prevent sarcopenia and excess body fat that predisposes patients to develop comorbidities such as diabetes and cardiovascular diseases. The results of this research will be useful to improve the comprehensive care of patients with CKD.

## Ethical considerations

The authors declare that all procedures followed were carried out in accordance with the principles of responsibility and ethics. Likewise, the research protocol was approved the by the ethics and research committees of the University of Guanajuato, and the Regional Hospital of High Specialty of Bajio; therefore, this research was developed in accordance with the principles established by the World Medical Association in the Declaration of Helsinki. Additionally, ethical principles of confidentiality were followed, and informed consent was obtained from all patients and research participants.

## Institutional review board statement

The research committee and the Research Ethics Committee of the Regional Hospital of High Specialty of Bajio approved this research, with the following registration numbers: CI/HRAEB/018/2022 and CEI-010-2022 It is declared that the procedures followed were carried out in accordance with the ethical standards of the Ethics and Responsible Research Committee, and in accordance with the provisions of the World Medical Association in the Declaration of Helsinki; that the workplace protocols on confidentiality and publication of patient data have been followed, and that informed consent has been obtained from the patients referred to in the article.

## Informed consent statement

Informed consent was obtained from all subjects involved in the study and is in the possession of the researchers.

## Data availability statement

The original contributions presented in the study are included in the paper; further inquiries can be directed to the corresponding author.

## Authors’ contributions

Piñon Ruiz MJ: Conceptualization; Methodology; Software; Formal analysis; Investigation; Resources; Writing-original draft; Writing-review & editing.

Huerta-Franco MR: Conceptualization, Methodology; Software; Formal analysis; Investigation; Writing-original draft; Writing-review & editing; Visualization.

Vargas-Luna FM: Software; Formal analysis; Visualization.

Apolinar-Jimenez E: Methodology; Software; Investigation; Supervision; Writing-original draft.

Soel Encalada JM: Validation; Resources; Writing-original draft.

## Conflicts of interest

The authors declare no conflicts of interest.
